# Xylooligosaccharides: A Bibliometric Analysis and Current Advances of This Bioactive Food Chemical as a Potential Product in Biorefineries’ Portfolios

**DOI:** 10.3390/foods12163007

**Published:** 2023-08-09

**Authors:** Tainá Manicardi, Gabriel Baioni e Silva, Andreza A. Longati, Thiago D. Paiva, João P. M. Souza, Thiago F. Pádua, Felipe F. Furlan, Raquel L. C. Giordano, Roberto C. Giordano, Thais S. Milessi

**Affiliations:** 1 Graduate Program of Energy Engineering, Federal University of Itajubá, Av. Benedito Pereira dos Santos, 1303, Itajubá 37500-903, MG, Brazil; 2 Graduate Program of Chemical Engineering, Federal University of São Carlos, Rodovia Washington Luíz, Km 235, São Carlos 13565-905, SP, Brazil; 3 Department of Chemical Engineering, Federal University of São Carlos, Rodovia Washington Luíz, Km 235, São Carlos 13565-905, SP, Brazil; 4 Institute of Natural Resources, Federal University of Itajubá, Av. Benedito Pereira dos Santos, 1303, Itajubá 37500-903, MG, Brazil

**Keywords:** xylooligosaccharides, lignocellulosic biomass, biorefineries, economic aspects, environmental impact, bibliometric analysis

## Abstract

Xylooligosaccharides (XOS) are nondigestible compounds of great interest for food and pharmaceutical industries due to their beneficial prebiotic, antibacterial, antioxidant, and antitumor properties. The market size of XOS is increasing significantly, which makes its production from lignocellulosic biomass an interesting approach to the valorization of the hemicellulose fraction of biomass, which is currently underused. This review comprehensively discusses XOS production from lignocellulosic biomass, aiming at its application in integrated biorefineries. A bibliometric analysis is carried out highlighting the main players in the field. XOS production yields after different biomass pretreatment methods are critically discussed using Microsoft PowerBI^®^ (2.92.706.0) software, which involves screening important trends for decision-making. Enzymatic hydrolysis and the major XOS purification strategies are also explored. Finally, the integration of XOS production into biorefineries, with special attention to economic and environmental aspects, is assessed, providing important information for the implementation of biorefineries containing XOS in their portfolio.

## 1. Introduction

Recently, consumer demands for safe and healthy food products have induced efforts towards the development of functional foods, such as prebiotics [1]. In this sense, non-digestible oligosaccharides such as xylooligosaccharides (XOS) have received great attention [2]. XOS are small oligomers (2–7 units) of xylose that have high added value due to their interesting prebiotic properties [3]. These oligosaccharides have been reported in a wide range of applications, such as anti-inflammatory, antioxidant, antitumor, and antimicrobial [2]. They are naturally present in honey, fruits, and vegetables, but not in sufficient amounts to have prebiotic effects or to be extracted by an economically feasible process [4]. In addition, the demand for XOS is rising due to the growing application of dry XOS probiotics in animal feed [5].

In this sense, lignocellulosic biomass emerges as a potential raw material for XOS production, due to its low cost and high availability. Biomass is mainly composed of cellulose, hemicellulose, and lignin, which results in around 70% of potential carbohydrates in its composition. Therefore, it is a valuable source of carbon for obtaining marketable products with high added value [6].

The use of biomass as a raw material to simultaneously produce different biofuels and other types of value-added products is the key concept of biorefineries, similar to petroleum-based refineries. The feasibility of using lignocellulosic biomass in biorefineries depends on the use of all components, i.e., cellulose, hemicellulose, and lignin fractions [6]. The alcoholic fermentation of glucose, the main component of cellulose, is a well-established process at industrial scale using the yeast *Saccharomyces cerevisiae*. However, applications for hemicellulose and lignin are still under development. Hence, both fractions are currently underutilized. In this sense, technologies that allow the use of hemicellulose are of great importance for the sustainable development of biorefineries [7], and the production of XOS from xylan can become an important destination for hemicellulose.

Biorefineries are emerging industrial facilities that include a wide range of technologies aimed at a sustainable and efficient transformation of all types of biomasses into bioenergy, multiple high-valued low-volume products, and chemicals [6]. Value-added products such as XOS produced together with primary products (biofuel and electricity) in the biorefinery represent a tactic that can reinforce the existing markets while creating new products and opportunities. In addition, one of the main technological challenges faced by biorefineries still resides in the full use of biomass at a competitive cost compared to established processes, which makes XOS production integrated to biorefineries an interesting approach to achieve the feasibility of second generation (2G) biofuels [8].

The market for XOS has been growing worldwide reaching USD 71 Million in 2022, and this market is expected to reach about USD 139 Million by 2032 [9]. XOS have an estimated compound annual growth rate (CAGR) of 7% [9] and Europe and North America are the main consumers [5]. The complexity of biomass processing requires research, process development, and innovation to be a viable technology.

In this sense, considering that there are still important gaps in the development of an efficient XOS production process from lignocellulosic biomass, such as the establishment of an appropriate biomass pretreatment method and conditions that lead to the solubilization of biomass hemicellulose in the form of oligomers, with minimal monomer production, feasible enzyme application, and process integration into biorefinery design with feasibility and low environmental impact. This review comprehensively discusses the technology of XOS production from lignocellulosic biomass with the aim of its application in integrated biorefineries. For the first time, a bibliometric analysis of XOS production processes is carried out, highlighting the main players in the field. In addition, the available pretreatment methods are critically discussed from a new point of view, using Microsoft PowerBI^®^ software to model and analyze literature data to identify important trends between different methodologies and process conditions (temperature, time, severity) to guide decision-making. Enzymatic hydrolysis and the main available XOS purification strategies are presented. Finally, the integration of XOS production in biorefineries, with special attention to economic data and environmental analysis impacts, is discussed, as there are few reports on the economic and environmental integration of XOS in biorefineries, providing important information for the establishment of biorefineries that include the co-production of XOS and other important products.

## 2. Xylooligosaccharides (XOS) Characteristics and Bibliometric Analysis

**Figure 1:**
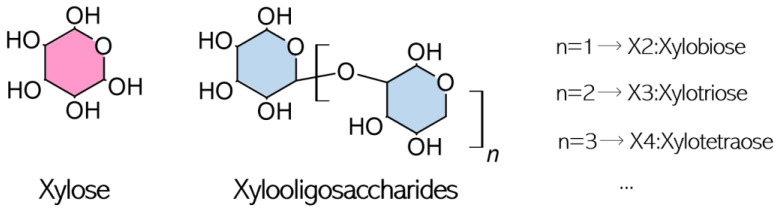
XOS main structure.

XOS can be obtained from the mild degradation of xylan, the most abundant component in the biome after cellulose. It can be found in different raw materials such as bamboo shoots, fruits, vegetables, wheat bran and straw, corn cobs, sugarcane residues, and rice straw [10]. These compounds consist of xylose units linked by β-(1,4) bonds (Figure 1) and may have several functional groups such as arabinosyl residues, acetyl groups, and uronic or phenolic acids [8], which mainly depends on the raw material of origin. The type of biomass used can also lead to different degrees of polymerization (DP), between two and seven xylose units, pattern of substitutions of the main chain and types of bonds [4,8]. These oligosaccharides have been receiving attention from the food and pharmaceutical sectors because they have several properties and beneficial health effects depending on their molecular distribution, although they are not digested in the human body due to their β bonds [11].

XOS have been associated with several health benefits, including gastrointestinal health and less flatulence in animals. A wide range of beneficial properties have been reported, such as anti-inflammatory, antioxidant, antitumor, and antimicrobial effects [5,12]. XOS have a high potential to be applied for human nutrition due to their physicochemical properties such as low viscosity, high water solubility, tolerance to high temperature, and acidic pH [2]. Due to these characteristics, XOS can pass through gastrointestinal enzymes and gastric acid, reaching the intestine and acting on the intestinal flora [13]. They can be used as dietary sweeteners, and as compounds in the formulation of drugs and food ingredients [14]. They have probiotic and organoleptic properties, increasing the digestion and absorption of nutrients, helping in calcium absorption and lipid metabolism, and acting as emulsifying agents, stabilizers, and fat substitutes [3,4,13,15]. According to Tang et al. [16], when metabolized by probiotic bacteria, XOS increase the production of small chain fatty acids, which maintain the integrity of the gastrointestinal protection once it regulates cecal cell proliferation and apoptosis. XOS also have important antimicrobial activities, as a wide range of clinically important bacteria have been reported as sensitive to XOS exposure [2]. In addition, XOS have been reported as promising agents in breast cancer prevention. Maeda et al. [17] observed that β-1,3-Xylooligosaccharides from green alga *Caulerpa lentillifera* induced apoptosis in human breast cancer MCF-7 cells. All these properties make XOS stand out when compared to other prebiotic compounds such as inulin, fructooligosaccharides (FOS), and galactooligosaccharides (GOS).

**Figure 2:**
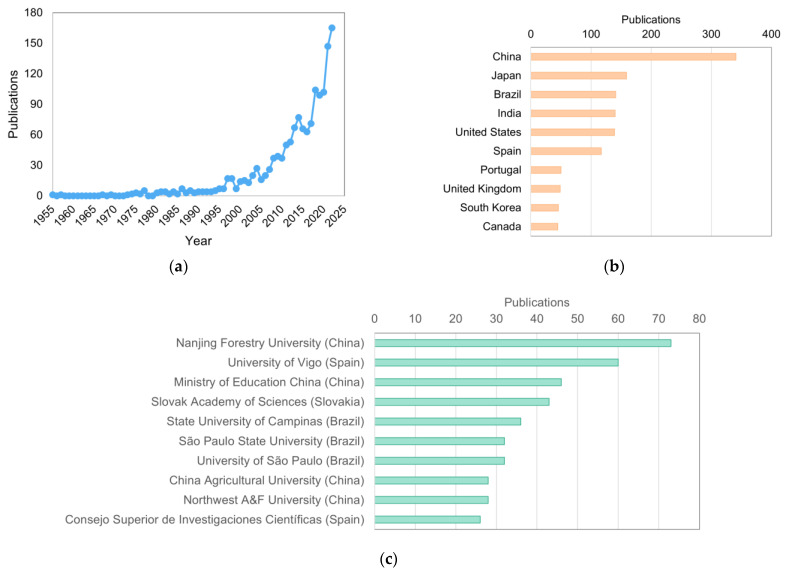
(**a**) Evolution on XOS publications over the years according to the Scopus database; (**b**) Most active countries on XOS research development; (**c**) Key institutions working with XOS innovation.

Thus, the interest in XOS has increased significantly in the last decades, especially xylobiose and xylotriose, since these xylooligosaccharides have the greatest prebiotic and sweetness properties when compared to XOS with higher DP [18,19]. A bibliometric analysis using the software VOSviewer^®^ (1.6.19) returned 1467 articles from the Scopus database, covering a wide range of topics related to the production, purification, and application of XOS. From 2012 to 2022, a significant increase in publications on XOS is noteworthy (Figure 2a), from 53 in 2012 to 165 articles in 2022, an increase of more than 210%.

**Figure 3:**
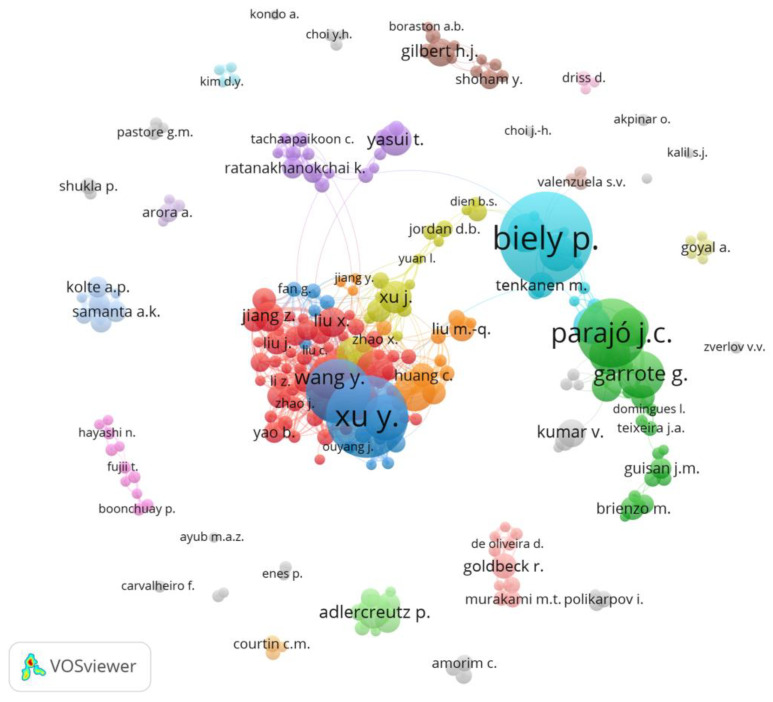
Most relevant authors in the XOS field; the bigger the circle, the higher is the author productivity. The colored clusters represent collaborative research groups.

When analyzing research trends, significant collaboration among different research institutions is observed, indicating a multidisciplinary approach in this field. The works predominantly focus on the areas of biochemistry, genetics and molecular biology, agricultural and biological sciences, and chemical engineering. In addition to the multidisciplinary nature, XOS research is relevant in different regions around the globe, with notable contributions from China, Japan, Brazil, and India (Figure 2b). Institutions include the Nanjing Forestry University (China), University of Vigo (Spain), and Ministry of Education China (China) (Figure 2c). In Figure 3, the most relevant authors in the XOS field are grouped according to the citations of their works, creating clusters that represent study groups on the subject. The authors with the most published works are P. Biely and Y. Xu, with 36 published articles each.

When it comes to funding institution, the National Natural Science Foundation of China have funded the highest number of works (158). However, Brazilian institutions stand out occupying second, third, and fourth place: National Council for Scientific and Technological Development (CNPq), São Paulo Research Foundation (FAPESP), and Higher Education Improvement Coordination (CAPES) have funded 85, 68, and 59 works, respectively. This is reflective of strong Brazilian polices and incentives for renewable energy and biorefineries development, since most of the Brazilian studies focus on the development of XOS production from agricultural waste and byproducts, such as sugarcane bagasse and straw.

The bibliometric analysis highlights the ongoing importance of XOS as functional ingredients and value-added products of industrial interest. Research in this field has focused on the development of efficient methods for XOS production, purification, and application, as well as on understanding their health benefits for humans. However, the high costs related to XOS production still is a limiting factor for its commercialization, making advances on the production process technology crucial to achieve greater efficiency and economic viability [4].

## 3. Lignocellulosic Biomass Composition and Availability

The potential of lignocellulosic biomass as feedstock for XOS production is closely linked to its chemical composition, which is mainly of cellulose, hemicellulose, and lignin [20]. Generally, lignocellulosic biomass is composed of 30–45% cellulose, 15–42% hemicellulose, and 10–30% lignin [21]. Cellulose is the most abundant component in biomass, being a linear polymer with amorphous and crystalline portions of *β*-glucopyranose unlinked by β-1,4-glycosidic bonds. The interaction between hydroxyl groups via hydrogen bonds in cellulose chains generates its crystalline structure with some amorphous portions, which restricts the access of catalysts to functional groups in the cellulosic chain, hampering both its solubilization and reactivity [22].

**Figure 4:**
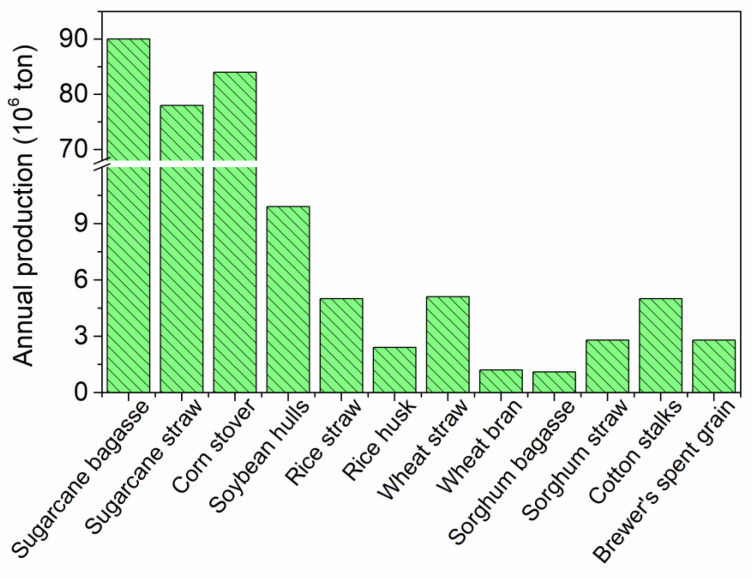
Availability of different LCB in the Brazilian scenario (according to data from Chandel et al. [6]; Aguiar et al. [23]; and Sganzerla et al. [26]).

Hemicellulose, in turn, is a branched heteropolymer composed mainly of xylan, which is a polymer rich in pentoses (such as xylose and arabinose), but can also present hexoses (D-glucose, D-galactose, and D-mannose), and acid units (acetic, D-glucuronic, and 4-O-methyl-D-glucuronic acid) in its composition [23]. Unlike cellulose, hemicelluloses are short, amorphous, and heavily substituted polymers, which facilitates their hydrolysis and solubilization [24]. Lignin is a rigid aromatic biopolymer composed of p-coumaryl, coniferyl, and sinapyl alcohols with a high molecular weight that links the other two fractions by covalent and hydrogen bonds [20]. As a consequence, a rather recalcitrant structure is formed, requiring a pretreatment step to release xylan, then allowing XOS production. From the biorefinery and circular economy point of view, the selective separation of biomass components is mandatory to produce value-added biomolecules. The use of lignocellulosic biomass for the production of XOS is an interesting approach since these materials are generally low-cost and do not compete with food [25]. In addition, it is a raw material with high availability, as can be seen in Figure 4 for the Brazilian scenario as an example.

## 4. XOS Production

**Figure 5:**
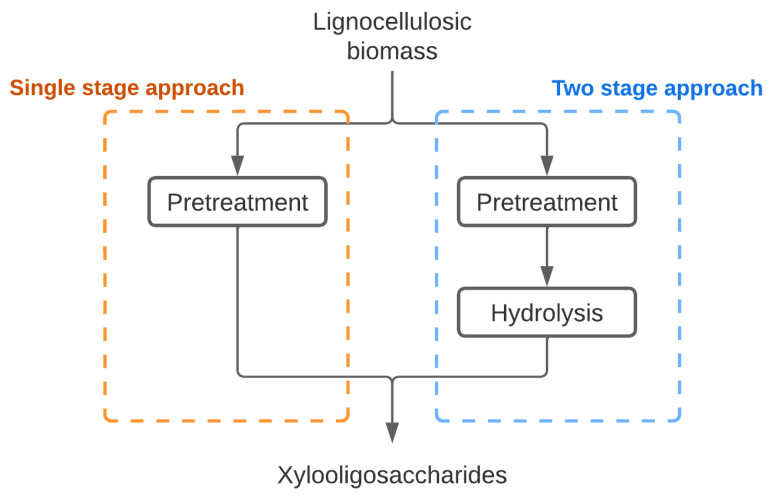
Strategies for XOS production from lignocellulosic biomass.

XOS are mainly produced through the hydrolysis of lignocellulosic biomass, specifically of its hemicellulose fraction. There are many challenges to be overcome in order to achieve a feasible production of XOS from lignocellulosic raw materials. Additionally, the design of the process is not trivial, since it depends on many factors, such as structural complexity, diverse composition, seasonality, and risk of deterioration [3,8]. There are different technologies available, but in general they can be classified as single-stage or two-stage approaches (Figure 5) [27]. In both strategies, the process starts with a pretreatment step to solubilize the xylan from the hemicellulose.

Considering that XOS with lower DPs have higher functional properties, and that hemicellulose is the fraction of biomass which solubilizes most easily [28], in single-step processes the severity of pretreatment necessary to achieve high biomass solubilization yields can lead to a high concentration of monomers [27], which is not desirable because this will impair the purification stage, as will be described in the next sections. In this sense, most of the reports use the two-step strategy, where the pretreatment is followed by enzymatic hydrolysis of the biomass [29]. In this case, xylanases will play a key role in XOS production. A summary is presented in Table 1, where it can be seen that the XOS yield (compared to the maximum potential XOS production considering the biomass hemicellulose content) can vary significantly between processes, according to the biomass type and the technology that was applied.

### 4.1. Biomass Pretreatments

**Figure 6:**
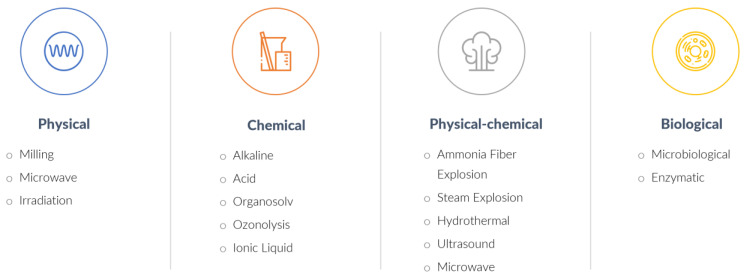
Biomass pretreatments methods (adapted from Aguiar et al. [23]).

The main challenge in biomass pretreatment for XOS production is to provide conditions that lead to high hemicellulose solubilization in the form of oligomers, with minimal production of xylose. There is a wide variety of pretreatment techniques that can be used to achieve this, which can be mainly classified as chemical, physical, physical–chemical, and biological methods (Figure 6) [23]. Milessi et al. [30] evaluated the production of XOS from sugarcane bagasse using different biomass pretreatments to extract xylan in the form of oligomers and observed that organosolv and hydrothermal methods stood out between the pretreatments studied, leading to the highest biomass deconstruction, XOS yield, and enzymatic hydrolysis conversion in the subsequent step, generating XOS with valuable probiotic and antimicrobial properties.

The hydrothermal pretreatment, also known as liquid hot water, is an efficient technique in terms of environmental, energy, and economic aspects [45]. This method is one of the most used for XOS production from lignocellulosic biomass, specially from sugarcane byproducts such as bagasse and straw [12]. Its principle involves the application of water or steam as a solvent at high temperatures and pressures. In this method, the biomass fibers expand, increasing the dissolution of hemicellulose [46]. Since it only uses pure water, without the addition of solvents, this pretreatment has been gaining prominence. It does not require the neutralization of an acid or recovery of a solvent, nor reactors resistant to corrosion, which reduces process costs [20]. This process is a self-hydrolysis of the biomass, due to the formation of hydroxonium ions [H_3_O^+^], breaking ester and ether bonds from the biomass structure. In addition, there is also the catalytic action of the acetic acid released from the deacetylation of the hemicellulose side chains [45]. Hydrothermal pretreatment is considered environmentally appropriate and has several advantages such as the low concentration of inhibitors produced, absence of chemical catalyst, increased contact surface of the resulting fiber, and hemicellulose solubilization [30]. After pretreatment, one can distinguish two phases: a solid fraction rich in cellulose and a liquor rich in hemicellulose.

The organosolv pretreatment is based on the use of organic solvents, such as ethanol, methanol, and acetone, to break lignin–lignin and carbohydrate–lignin bonds, causing the removal of lignin and consequent deconstruction of the lignocellulosic structure [47]. Considering integrated biorefinery plants, the use of ethanol as a solvent is interesting, since it is available in the plant [48]. The use of the organic solvent–water mixture at high temperatures allows the solubilization of lignin in the organic phase, while the aqueous phase operates as a hydrothermal treatment, thus carrying out the simultaneous pre-hydrolysis and delignification of the biomass [49]. With the removal of lignin and solubilization of hemicellulose, the surface area of the resulting solid cellulosic fiber increases considerably, facilitating the access of hydrolytic enzymes and increasing efficiency in the release of fermentable sugars [50]. However, this method has high cost and needs a step for solvent recovery [51].

The alkaline and acid pretreatments are other methodologies extensively studied to break the biomass structure. Despite generally leading to the formation of monomers, the use of mild conditions could allow the application of these techniques for XOS production. In addition, the use of organic acids as replacements of inorganic acids are described as a strategy to reduce the formation of byproducts, promote less device corrosion, and achieve XOS production in a desired range of DP. Cheng et al. [52] obtained a XOS yield of 58.7% from sugarcane bagasse using syringic acid pretreatment (180 °C, 20 min, and 9% *v*/*v* of acid). However, xylose was the main byproduct observed by the authors. Considering this, a two-step approach with a lower XOS yield but with a high availability of xylooligomers (more than seven xylose units) is preferable to a one-step approach with a high XOS yield but with the formation of high monomer titers. The xylooligomers can be hydrolyzed to XOS in the sequential hydrolysis step, whereas monomers will harm the purification step.

Although there is a variety of pretreatment techniques that can be used for XOS production, no efficient and well-established pretreatment for this purpose is applicable in all situations. In addition, each method has advantages and disadvantages, related to their yield and to the generation of impurities. Concerning environmental impacts, a sound analysis is demanded for the selection of the best method for each case.

Since the release of hemicellulose in the form of xylooligomers will depend on the pretreatment methodology, severity, and on the biomass used, an extensive literature research was put forth, treating the data with Microsoft Power BI (2.118.621.0), aiming to identify important correlations and factors that can lead to higher XOS yields. A selection of articles was carried out in three stages: (1) selection of articles about XOS from the Scopus, Google Scholar, and ResearchGate databases; (2) analysis of the articles to remove bibliographic reviews and keep only research papers with original results; and (3) in-depth analysis and classification of the methodology used and the results obtained.

**Figure 7:**
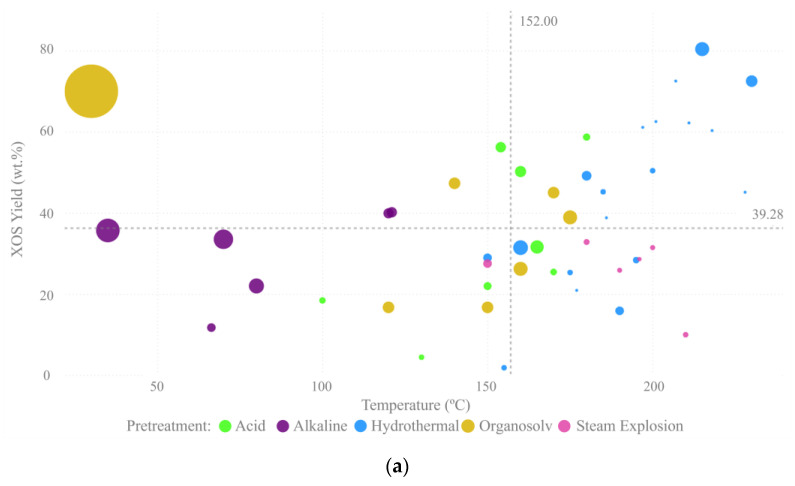

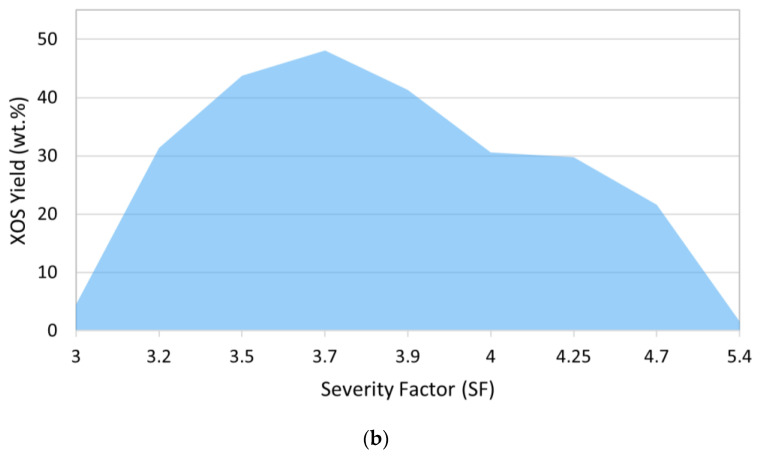
XOS yield tendencies created with Microsoft Power BI (**a**) XOS yield variation with temperature for different pretreatment methodologies, where bigger circles indicate longer processes; (**b**) XOS yield variation according to the severity factor of a pretreatment.

In Figure 7a, it is possible to notice that hydrothermal and organosolv treatments presented the highest XOS yields. Alkaline pretreatments, together with lower reaction temperatures, resulted in lower yields of xylooligomers, whereas hydrothermal treatments used higher temperatures and achieved higher yields. Acid pretreatment, besides the use of high temperatures, did not achieve high XOS yields. As mentioned before, hemicellulose is highly susceptible to acid hydrolysis and the acid pretreatment conditions generally lead to high solubilization of this fraction in the form of monomers, decreasing the XOS production [23]. In general, short pretreatments, especially together with low temperatures, leads to the lowest yields.

In this sense, the main tendency of the analyzed data is that higher temperatures require shorter times to achieve the same XOS yield than lower temperatures with longer process times. The influence of this binomial temperature:time reflects the strong effect that the pretreatment severity can have on the XOS production yields. The severity factor (*SF*) of a pretreatment is calculated according to Equation (1) [53], which represents a continuous profile of temperature versus time, where t is the residence time, *T_i_* is the temperature at some instant, and *T_Ref_* is the reference temperature (100 °C). The higher the *SF,* the more severe the conditions of the pretreatment applied.

(1) $$SF=log\sum_{i=1}^{n}\left[t\times \exp\left(\frac{T_{i}-T_{Ref}}{14.75}\right)\right]$$

As it can be seen in Figure 7b, XOS yield increases with severity factor up to *SF* around 3.7. Above this *SF*, the conditions to disrupt the biomass structure are too strong and reduce XOS titers, by the formation of monomers instead of xylooligomers or the production of inhibitory compounds by degradation of glucose, xylose, and lignin, such as hydroxymethylfurfural (HMF) and furfural. Milessi et al. [30] observed a 16-fold increase in the formation of furfural by increasing the *SF* from 4.5 to 5.4 in the hydrothermal pretreatment of sugarcane bagasse. In this sense, this tendency graph can show a pattern between *SF* and XOS yield that can help in decision-making concerning the conditions for the pretreatment of biomass, since it includes different biomasses and pretreatment types. Figure 7b indicates that in pretreatment projects, a *SF* around 3.7 is desired to avoid too weak conditions that do not efficiently solubilize the hemicellulose and too strong conditions that lead to the production of monomers and inhibitors instead of oligomers.

### 4.2. Enzymatic Hydrolys

The method used for XOS production will directly interfere in the DP obtained at the end of the process and the functional properties of the XOS are directly related to DP. Industrially, DPs between 2 and 6 are considered the most interesting. Therefore, the hydrolysis of the xylooligomers produced in the pretreatment must occur in a controlled and accurate way [10].

The enzymatic hydrolysis of xylooligomers is catalyzed by endo-xylanases (EC 3.2.1.8), that act in the middle of the xylan chain, breaking 1,4-β-D linkages [54]. These enzymes can be obtained from a variety of organisms, such as fungi, yeasts, and bacteria [27]. The choice of the xylanase is extremely important to maximize the production of XOS and minimize the production of xylose, and it is important to consider the activity of endoxylanases, resistance to metal ions, optimal pH, and reaction temperature [55].

Due to the high complexity and heterogeneity of xylan, there is a range of xylanases with different specificities and primary sequences. Xylanases are classified not only in EC 3.2.1.x number, but also in families 5, 7, 8, 16, 26, 43, 52, and 62. Each of these families is characterized by particular catalytic mechanisms [56]. In Table 1, it is possible to see some works using different xylanases to produce XOS from different biomasses through a two-stage production strategy. Due to the complexity of lignocellulosic biomass, some accessory enzymes can be used together with xylanases to increase hydrolysis conversion, such as arabinofuranosidases and feruloyl esterases [13].

The high cost of enzymes is one of the major challenges in implementing this process on an industrial scale. Much effort is being made for reducing the cost of enzymes for XOS production, such as the improvement of their activity and enzyme immobilization, which can improve the operational stability of the enzyme, facilitate the separation and recovery from the reaction medium, and allow its recycling in consecutive batches [57]. Alagös et al. [58] studied the immobilization of a xylanase from *Thermomyces lanuginosus* in carbon nanotubes activated with glutaraldehyde and observed a 24-fold increase in enzyme thermal stability. Nascimento et al. [59] immobilized an endoxylanase from *Thermomyces lanuginosus* PC7S1T in a calcium alginate gel allowing the enzyme reusability for 11 cycles. Milessi et al. [60] achieved a remarkable 8600-fold increase on enzyme stabilization through the immobilization of endoxylanase from *Bacillus subtilis* in agarose-glyoxil. All these results highlight how enzyme immobilization may be an effective tool to achieve the feasibility of XOS production from biomass.

### 4.3. XOS Purification

Essentially, XOS purification processes include the fractionation of XOS chains into a range of DP and the separation of XOS from other undesirable compounds [61]. In addition, for food applications, interest usually lies in relatively low PD products (mainly, PD 2-6) and without monosaccharides [62]. In fact, the presence of glucose and xylose changes the calorific value and sweetness power of XOS mixtures [14]. In this sense, to produce food-grade XOS from biomass, it is necessary to remove monosaccharides and non-saccharide compounds from the hydrolysate rich in XOS, in order to obtain a concentrate with high XOS content, since commercial XOS purity must be in the range 75–95% [63].

The composition of XOS and the presence of undesirable products in the post-pretreatment crude liquor, depend on the source of biomass and on all process steps necessary to achieve the depolymerization of hemicellulose. For example, there is a high production of undesired compounds during the pretreatment, including acetic acid, furfural, and HMF. Alkaline pretreatment can also yield soluble lignin in the crude XOS mixture [64]. The prebiotic biological function depends on the purity of the product [65]. Although there is no evidence of negative effects of these compounds on human health [61], a deleterious impact of acetic acid, furfural, and dissolved lignin derivatives on the XOS prebiotic effect has been reported [66].

The XOS production through a two-step processes, i.e., pretreatment followed by enzymatic hydrolysis, generally yields less undesired compounds, consequently simplifying the purification step and reducing costs [67]. A general and ready-to-use purification strategy for XOS production is not realistic, due to the many available sources of lignocellulosic materials and all the possible upstream processes. Nevertheless, there are many applicable guidelines and a number of important experimental results. Table 2 provides a summary of the XOS purification processes through several techniques, sometimes combined in sequential order, with observations about the findings from each study. Generally, XOS purification is mainly carried out by adsorption separation, solvent extraction, membrane separation, and chromatographic separation [14]. Acetic acid and other volatile components may be removed by vacuum evaporation or spray drying [61,62]. These operations are also useful for the overall concentration and off-flavors reduction [61].

Solvent extraction can be useful in the XOS purification strategy, especially for the removal of non-saccharide components [68,69,70,71,72]. Ethyl acetate is usually in the organic phase, resulting in a selectively refined aqueous phase and a solvent-soluble fraction with a variety of undesired extractive- and lignin-derived compounds (such as low molecular phenolics, waxes, fatty acids). After organic extraction, precipitation in the presence of water-soluble organic solvents and solvent extraction of freeze-dried solids were applied to further decrease the content of nonvolatile components other than XOS [69,70,71,72], but it is not clear whether the methods are technically or economically attractive. Moreover, results in the precipitation frequently indicate low improvement of purity or limited XOS recovery, depending on the solvent applied [69,70,71,72].

Membrane technology is a promising method for refining and concentrating XOS [73,74]. It is a technique that leads to highly concentrated XOS with low energy requirements and ease of operation [14]. While effective to separate XOS from higher molecular weight compounds, ultrafiltration may fail to remove monosaccharides and other undesirable compounds with low molar mass [75,76]. Consequently, a combination of ultrafiltration and nanofiltration is a possible approach to achieve the desired purification [77]. Despite that, there are reports of effective separation of XOS from monosaccharides in a two-step ultrafiltration process [75,78]. Swennen et al. [72] compared ultrafiltration and ethanol precipitation for the fractionation of arabinose-substituted XOS from wheat and observed that the ultrafiltered XOS were more heterogeneous and poly-disperse. Wijaya et al. [79] applied two steps of nanofiltration in polyvinylidene fluoride ultrafiltration membranes of 150,000 Da and 600–800 Da and achieved xylobiose with a purity of 90.1% (41.3 g/L xylobiose and 4.1 g/L of xylose) from an empty fruit bunch.

Separation by a solid agent has been evaluated for XOS purification, which includes many different configurations, from batch adsorption to chromatographic protocols. Many adsorption agents have been used, such as activated charcoal, acid clay, bentonite, diatomaceous earth, aluminum hydroxide or oxide, and silica [61]. Treatment with activated charcoal was reported to be a feasible option for the removal of extractives-derived, lignin-derived, and carbohydrate-degradation compounds present in XOS mixtures [14]. On the other hand, ion-exchange resins have been employed, coupled with other purification strategies, to remove salts, heavy metal ions, negatively or positively charged organic compounds, and pigments [67]. Milessi et al. [30] used activated charcoal in the purification of XOS from sugarcane bagasse and achieved a nearly complete (~100%) removal of furfural and HMF. However, this strategy also caused the adsorption of around 50% of XOS on the activated charcoal. Chen et al. [80] developed batch adsorption studies with activated carbon and ethanol–water elution to recover 47.9% (*w*/*w*) of XOS produced from *Miscanthus* × *giganteus* (M × G), a warm season perennial rhizomatous grass being developed as a bioenergy crop for the production of fuel. XOS could be fractionated by DP according to the ethanol concentration. However, the collected fractions did not show the desired prebiotic function. Only after a sequence of ion exchange resin treatments in order to remove heavy metal ions, negatively charged organic compounds, and pigments, the product showed comparable effects with commercial sources of XOS, reaching 74.9% (*w*/*w*) xylose oligomers, with a DP between 2 and 6.

**Table 2:** Summary of XOS purification processes described in the literature, where IE: ion exchange (solid phase separation); IEC (−): ion exchange chromatography (anion); IEC (+): ion exchange chromatography (cation); GFC: gel filtration chromatography; SE: solvent extraction; GFC: gel filtration chromatography; ACA: activated carbon adsorption; MS: membrane separation; UF: ultrafiltration; NF: nanofiltration; SP: solvent precipitation; FDSE: freeze-drying-solvent extraction.

XOS Source	Downstream Operations	Observations	Ref.
Brewer spent grain	SE/SP or FDSE or IE	SP with ethanol presented limited yield SP with 2-propanol or acetone presented similar results FDSE presented no significant purification effect	[71]
Wheat Flour	MS	Focused on arabinoxylooligosaccharides One or two MS procedures applied to reach different fractionation	[72]
Rice Husk	MS/IEC (−)/IEC (+) or MS/SE/IEC (−)/IEC (+)	SE is proper to remove non-saccharide compounds Better purification when applying SE before IEC	[68]
Almond shells	Spray drying MS	Membrane performance with different molecular cut-off (1, 2.5, 3.5, and 8 kDa) at pressures between 2.6 and 9 bar Favorable separation from lignin-related products at low fluxes of permeate and with membranes of “low cut-off” Not much irreversible fouling effect after 2 to 4 h of continuous use	[62]
Rice straw	GFC	Separation of different byproducts, including di-, and monosaccharides (from oligosaccharides of DP ≥ 3 to high DP oligosaccharides, DP ≥ 23)	[81]
Oil palm empty fruit bunches	GFC	Purity higher than 74% and at least 83% consisted of XOS	[82]
Corn stalk	Acid precipitation/ ACA (ethanol elution)	Focused on color (chromophore) Highest purity of XOS (97.9%) using 30% ethanol eluate Six different activated carbons tested, purity of XOS increased to 87.28% from 67.31%, and the color value decreased to 1050 from 4682	[83]
Poplar wood chips	Calcium hydroxide treatment followed by ACA	Increase of AC dosage has the tendency to adsorb more XOS with DP > 6 than XOS with DP between 2 and 6 Removal of lignin derivatives (66.9%) and furfural (70.1%) pH during the ACA influences lignin removal Moderate loss of XOS	[84]
Bamboo	ACA or UF/IE/NF	Purity of 92.3%, and a total sugar proportion as high as 98.9% Protein removal rate of 77.7%, sugar loss rate of 2.0%, and decolorization rate of 52.3%	[85]
Sugarcane bagasse	ACA	Efficient removal of furfural and hydroxymethylfurfuralLow XOS yield obtained (~50% XOS adsorption together with contaminants)	[30]
Oil palm empty fruit bunches	Enzymatic polymerization to remove phenolic compounds from crude XOS and MS	50.2% of the total phenolic compounds polymerized and precipitated, additional 22.6% removed by MS For feasibility, enzymatic polymerization demands research in enzyme reuse technology	[86]

Chromatography is an alternative that has the advantage to yield XOS with high purity and separated by molecular weight or chemical structure [81]. Moniz et al. [81] applied gel filtration chromatography to fractionate the autohydrolysis product of rice straw into diverse classes of XOS based on the DP, and separated from di-, monosaccharides, and other similar sized byproducts. Ho et al. [82] applied gel filtration chromatography in the XOS production from empty fruit bunches, which is a residue formed during palm oil production. As a result, the overall purity was higher than 74%. Wang et al. [87] obtained a XOS from wheat bran with 75% purity by applying a combined strategy which involved the discoloration of hydrolysate using anion exchange resin D392 and a cation-exchange resin to remove impurities. Álvarez et al. [88] produced a XOS with 81% purity from steam-exploded barley straw using size-exclusion chromatography. However, the chromatography is usually performed to obtain purified fractions of XOS for structural characterization and analytical quantification, due to the difficulty for scale up [63].

As each process has advantages in some aspect, it seems that a reasonable approach to purify XOS may result from a combination of different techniques. As an example, Vegas et al. [68] used a combination of nanofiltration, solvent extraction, and ion exchange chromatography to achieve 90.7% XOS purity. Jiang et al. [85] developed a purification process including membrane ultrafiltration, ion exchange desalination, and monosaccharide removal by nanofiltration. The XOS source was a crude xylan solution extracted from bamboo. The protein removal was 77.7%, sugar loss was 2.0%, and decolorization was 52.3%. The final XOS product was obtained with 7.5% yield and 92.3% purity. As previously mentioned, many approaches were reported to purify XOS produced from lignocellulosic biomass. However, the choices and the optimization of the processes are far from being a closed topic. Moreover, the process synthesis and development should certainly take the biomass source and upstream process into account, but also consider the economic and environmental aspects.

## 5. XOS in Biorefinery Platforms and Market Opportunities

### 5.1. XOS Production in Biorefineries

The understanding of the role of biorefineries in the economy is consistent with leading energy organizations such as the International Energy Agency (IEA), which provided the most used definition “*biorefinery: the sustainable processing of biomass into a spectrum of marketable products (food, feed, materials, chemicals) and energy (fuels, power, heat)*” [89], and the National Renewable Energy Laboratory (NREL) “*A biorefinery is a facility that integrates biomass conversion processes and equipment to produce fuels, power and (organic) chemicals from biomass*” (NREL, 2011). Therefore, this concept is similar to that of the conventional petroleum refinery, which refines crude oil into products that can be used as fuels for transportation and electricity generation, and as high-value chemicals [90].

Bioenergy comprises low-value but high-volume biofuels, commodities such as biodiesel, bioethanol, or biogas, as well as bioelectricity and process bioheat. Bioproducts can be high-valued, obtained in low-volume, but they can also be intermediate molecules, building blocks, which are feedstock for further processing. They include biopharmaceuticals, biocosmetics, bionutrients, biochemicals, biofertilizers, and biomaterials. High-value bioproducts are designed to increase the profitability of biorefineries. On the other hand, high-volume biofuels, biopower, and bioheat produced in situ reduce energy costs for internal use and provide additional revenue. High-value bioproducts in particular are attracting commercial attention because innovative technologies can produce some of them at a reasonable cost [91].

**Figure 8:**
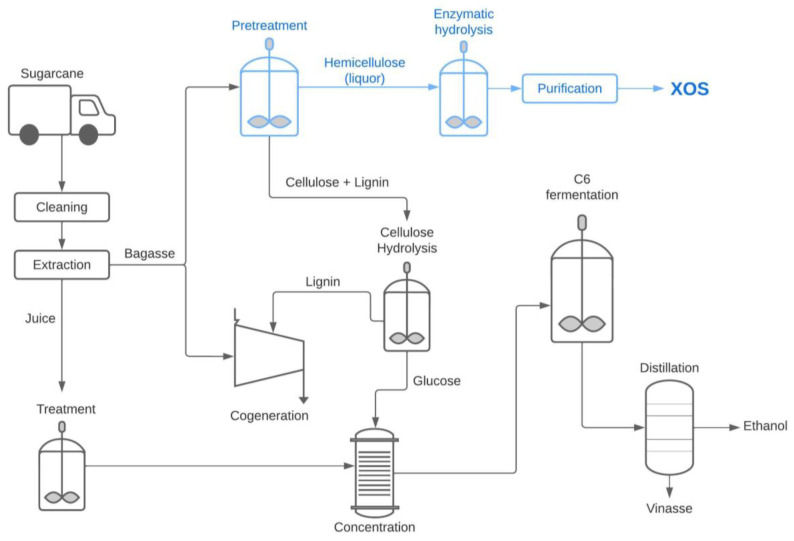
Integrated sugarcane-based biorefinery for the co-production of bioethanol, XOS, and energy applying the two-stage XOS production (in blue).

In the context of sugarcane biorefineries, hemicellulose can be fractionated and recovered from lignocellulose. This creates an opportunity to selectively fractionate hemicellulose directly into a marketable product, which enables the inclusion of high-value compounds such as XOS from sugarcane lignocellulosic byproducts in the portfolio of biorefineries [92]. Pereira et al. [93] reported an integrated biorefining strategy of sugarcane bagasse to selectively fractionate it into its main constituents, and to allow the production of high-value products (XOS from hemicellulose, lignin nanoparticles from lignin, and cellulose nanofibrils from the unhydrolyzed cellulosic solid residue) alongside ethanol at a high yield. Milessi et al. [30] studied the whole sugarcane bagasse XOS production chain, starting with biomass pretreatment, enzymatic hydrolysis of the hemicellulose fraction, purification, and evaluation of nutritional properties of the final product. Valladares-Diestra et al. [94] applied the biorefinery concept in the synergistic production of xylanases and other enzymes (such as proteases), and the application of enzyme complexes in XOS production, through enzymatic hydrolysis of xylan from sugarcane bagasse. Figure 8 shows the insertion of the XOS production in a sugarcane biorefinery.

### 5.2. XOS Global Market and Opportunities

**Figure 9:**
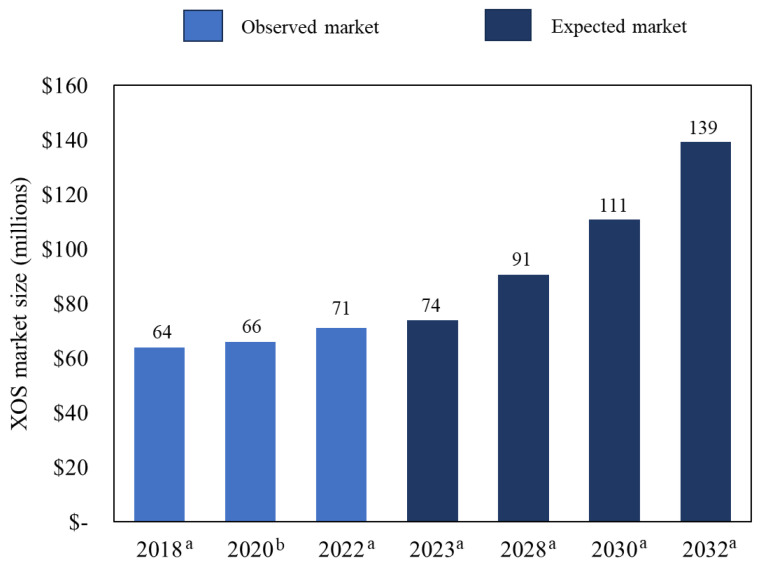
Market size of XOS. Data source: ^a^ FMI [9] and ^b^ AR [95].

The commercialization of bioproducts with high value depends on market demands, production costs, and avoiding competition with fossil-fuel-derived products. Therefore, the most promising bioproducts combine market volumes with medium to preferably high selling prices. In this context, XOS represent a promising product for the valorization of sugarcane biorefineries, since it is a bioproduct with a strong market growth (Figure 9), an increasing market demand (XOS market CAGR expected of 7% from 2023 to 2033, [9]), and high value for sales (Table 3).

The market demand was expanding at a CAGR of 2.7% from 2018 to 2022. However, the market is expected to grow at a faster rate (CAGR of 7%). This is mainly due to the expansion of the livestock sector and packaged food, along with the increasing demand for natural ingredients in animal feed [9]. As seen in Table 3, the prices range from USD 20.00/kg of XOS to USD 20,000.00/kg, depending on its purity. The main XOS product specifications in the market are 95% XOS powder, 70% XOS powder, 35% XOS powder, 20% XOS powder, and 70% XOS syrup [95].

The key players operating in the XOS market are: Anhui Elite Industrial Co., Ltd. (Hefei, China), Dongguan ALL Natural Plant Extracts Co. (Xinsheng, China), Longlive (Jinan, China), Jiangsu Kangwei Biotechnology Co., Ltd. (Nanjing, China), Injiang Aksu Hengfeng Sugar Co., Ltd. (Aksu, China), Henan Shengtai Biotechnology Co., Ltd. (Zhengzhou, China), Yibin YAatai Biotechnology Co. (Yibin, China), YuHua Group (Guangzhou, China), Henan Yuanlong Biotechnology Co., Ltd. (Nanjing, China), Shandong Bailong Chuangyuan Bio-tech Co., Ltd. (Dezhou, China), Van Wankum Ingredients (Maarssen, The Netherlands), Shandong Fengyuan Zhongke Ecological technology Co., Ltd. (Zaozhuang, China), Hebi Taixin Science & Technology Co., Ltd. (Hebi, China), Suntory Holdings Ltd. (Osaka, Japan) [95,102,103]. The United States held the highest market share of XOS consumption, followed by Germany and Japan, accounting for 34%, 13%, and 1.5% of the XOS market, respectively [9]. It is projected that the United States will remain dominant during the next years because of the rising health awareness among consumers.

### 5.3. Techno-Economic and Life Cycle Assessment of XOS Production Integrated into Biorefineries

As previously stated, XOS are of great interest to various industries and represent a promising product for valorizing biorefineries. Nevertheless, the production of XOS requires large amounts of investment and costly and specialized equipment.

There are a large number of possible combinations of feedstock, pretreatment options, conversion technologies, and downstream processes. Moreover, production of XOS generates unwanted products such as furfural and hydroxymethyl furfural in the mixture which can increase even more the purification costs (capital and operational) of this bioproduct. All these options of pathways for XOS production make the comparison of alternatives difficult. Therefore, the evaluation of XOS technologies from the technical, economic, and environmental perspective is mandatory for the implementation of its large-scale production in biorefineries. Studies that performed this techno-economic–environmental analysis (TEEA) are scarce in the literature.

Lan et al. [104] assessed the economic performance of a biorefinery producing XOS from *Miscanthus* by autohydrolysis based on a simulation developed in ASPEN Plus. The analyses of the authors were carried out in varying biorefinery capacities (50–250 oven dry metric ton (ODMT)/day) and levels of XOS content (80%, 90%, and 95%). The minimum selling price found for the product (XOS MSP) varied between USD 3430 and USD 7500 per metric ton (MT) for 80% content of XOS, between USD 4030 and USD 8970 per metric ton (MT) for 90% content of XOS, and between USD 4840 and USD 10,640 per metric ton (MT) for 95% content of XOS. The results showed that increasing the biorefinery capacity implied a significant reduction of XOS MSP. Higher purity led to higher XOS MSP, due to lower yields and higher operating and capital costs.

Swart et al. [105] carried out the techno-economic analysis (TEA) of the valorization of brewers’ spent grains (BSG), used as raw materials for the production of xylitol and XOS. They evaluated the application of hydrothermal processing using high solids in a xylitol and XOS-production biorefinery annexed to a brewery. The authors assessed three scenarios for the production of: (A) the sugar replacement xylitol; (B) XOS; and (C) co-production of xylitol and XOS. The economic evaluation was performed comparing capital and operating expenses from process simulations implemented in Aspen Plus. The authors found a positive economic performance for all scenarios: the internal rate of return (IRR) values obtained were greater than the hurdle rate of 9.7% for all scenarios when considering a conservative market price for xylitol and XOS of USD 4500/t. They also found that dedicated production of XOS was economically more favorable with a minimum required selling price (MRSP) of USD 2509/t for XOS, which is nearly half that of xylitol (USD 4153 t of xylitol). Furthermore, the xylitol and XOS co-production scenario reached the lowest MRSP, of USD 2182/t. It has been shown that byproduct revenue contributions in multi-product scenarios support the economic feasibility of the concept of a small-scale biorefinery attached to a brewery, since the byproducts contributed to 32.7%, 14.2%, and 27.5% of the revenue generated in scenarios A, B, and C, respectively.

Cao et al. [106] conducted the TEA of four different organic acids, including formic acid, glycolic acid, lactic acid, and acetic acid for XOS production. The authors estimated the cost of producing one-ton XOS by these different acids. The authors found that the maximum cost of the process is associated with the raw materials, which is about 38% of the production cost for one-ton XOS. The main difference between the four different acid hydrolysis processes lies in the cost of pretreatment and conditioning, in which the ratio of capital recovery charge is 11%, raw material is 2%, process electricity is 0.8%, and the fixed cost is 3% of the total product cost. For each ton of XOS produced, the order of the acids in terms of the combined cost of raw materials and process electricity was as follows: glycolic acid > lactic acid > formic acid > acetic acid. Considering the production cost of the factory, acetic acid should be preferred as the catalyst.

Sganzerla et al. [107] evaluated the TEA of XOS production from BSG in a single and two sequential flow-through subcritical water hydrolysis (SWH) reactors at different temperatures (80 and 180 °C). The lowest cost of manufacturing (18.36 USD/kg of XOS) was obtained for the process with two sequential reactors. Although the fixed capital investment was 0.82-fold lower for the process with a single reactor, the gross profit of the process with two sequential reactors was 30% higher, which resulted in a return on investment of 54.26% and a payback of 1.84 years. In conclusion, SWH is a potential candidate process to produce XOS from BSG.

Heerden [108] performed the TEEA of the annexed production of XOS from sugarcane in a South African sugar mill. The economic benefits and the greenhouse emissions (GHG) of two scenarios were investigated: 2G XOS production (XOS-2G scenario, 1G2G, and XOS production) and integrating 1,3-Propanediol (PDO) with XOS production (PDO-XOS scenario, 1G2G PDO, and XOS co-production). The results showed that for a XOS market price of USD 25/kg, the internal rate of return (IRR) was calculated to be 87.94% and for a minimum acceptable rate of return of 20%, a MPSP of USD 5.50/kg was estimated for the 1G2G XOS scenario. The addition of PDO production, in the PDO-XOS scenario, increased both the capital and operating costs of a XOS biorefinery. Therefore, XOS production in both scenarios was an excellent candidate for a diversification of the products portfolio in that sugarcane biorefinery, with a promising economic performance. The GHG emissions were estimated as 9.21 kg CO_2eq_/kg XOS and 11.39 kg CO_2eq_/kg XOS in scenarios XOS-2G and PDO-XOS, respectively.

A comparative TEEA study was carried out by Lopes et al. [109] considering lignocellulosic-based small-scale biorefineries, integrated with a piggery waste-based anaerobic digestion platform, located in Portugal and Chile. The production of isobutene using a genetically engineered *Escherichia coli* coupled with the removal and purification of XOS, obtained after a feedstock hydrothermal pretreatment, was assessed using Aspen Plus. Corn stover was used in the Portuguese case study and wheat straw in the Chilean case study. The results showed that the isobutene/XOS biorefinery was economically viable both in Portugal and in Chile, mainly due to the high market value of XOS. The biorefinery had lower production costs for isobutene and XOS (1 USD/kg of isobutene and 1.18 USD/kg of XOS) when located in Portugal, compared to Chile (1.14 USD/kg of isobutene and 1.56 USD/kg of XOS). In comparison with the market prices adopted by the authors in the study for both products (isobutene: 2.02 USD/kg; XOS: 4.05 USD/kg), the profit margin can be high, especially for XOS. However, it is important to note that these production costs do not include capital expenditures, so the minimum selling price of these products is likely higher than indicated. Concerning the environmental analysis, the biorefinery led to a lower environmental impact of GWP emissions when located in Chile (48.8 kgCO_2eq_/GJ_isobutene_, compared to 60.7 kgCO_2eq_/GJ_isobutene_ in Portugal).

Barbosa et al. [110] assessed the economic and environmental perspective of the co-production of cello-oligosaccharides (COS) and XOS from sugarcane straw (COS is the main product and XOS the byproduct). Regarding the capital expenditures of their most optimistic scenario, the higher fraction (78%) was related to the COS production stage, followed by the XOS production stage (12%), and sugarcane pretreatment (~10%), indicating that the costs related to XOS purification (chromatography costs) tend to be higher than the costs related to sugarcane straw pretreatment in higher scales. Regarding the environmental analysis, using the life cycle assessment approach (LCA), XOS’ GWP ranged from 3.8 to 5.5 kgCO_2eq_/kg of XOS.

The LCA of the bioethanol and XOS co-production from the lignocellulosic residue barley straw from cereal cultivation and BSG was carried out by González-García et al. [111]. LCA results identified two environmental hotspots over the whole biorefinery chain: the production of steam required to achieve the high autohydrolysis temperature (responsible for contributions higher than 50% in categories such as acidification and global warming potential) and the production of enzymes required in the simultaneous saccharification and fermentation (>95% of contributions to terrestrial and marine aquatic ecotoxicity potentials). Since enzyme production involves high-energy intensive background processes, the most straightforward improvement challenge should be focused on the production of steam. The authors also estimated the global warming potential, an important category nowadays. They found emissions of 4.21 kg CO_2eq_/kg of XOS.

For comparison and visualization purposes, Table 4 and Table 5 summarize the economic and environmental performance of the XOS studies found in the literature. However, besides the great potential of XOS stated by all the economic and environmental analyses, some key challenges still need to be overcome to allow production in large-scale biorefineries, such as formulation, stability, flavor and taste, and consumer acceptance [13].

## 6. Conclusions

XOS are an interesting bioactive food chemical, to be included in the portfolio of biorefineries for valorization of the biomass hemicellulose fraction. These compounds have interesting properties for food products, cosmetics, and pharmaceutical industries, and an increasing market size, especially xylobiose and xylotriose that have greater functional properties than XOS with higher DP. However, several challenges must be addressed to allow the implementation of a feasible and economically viable XOS industrial processes. Despite economic and environmental analyses indicating how XOS can become a significant high-value, low-volume bioproduct in the portfolio of biorefineries, the diversity of biomass sources and process routes complicates a direct technical and economical comparison, with minimum selling prices varying significatively with raw material and integrated biorefinery scenarios. Nevertheless, general approaches and conclusions are scattered throughout the scientific and technological research over the decades. Various pretreatment methods have been explored aiming at XOS production from lignocellulosic biomass. It is clear that the severity of the pretreatment plays an important role in ensuring high hemicellulose solubilization with minimal formation of monosaccharides. SF values around 3.7 are desired to avoid too weak conditions that do not efficiently solubilize the hemicellulose and too strong conditions that lead to the production of monomers and inhibitors instead of oligomers. From an economic and environmental point of view, the use of mild conditions is strategical since it requires less energy demands and generally presents lower environmental impacts. The two-stage approach for XOS production (pretreatment to the xylooligomers extraction followed by enzymatic hydrolysis) leads to higher yields and makes the purification steps easier, and the application of immobilized enzymes can allow the achievement of process economic feasibility by decreasing enzyme costs in the process. The purification of XOS from lignocellulosic hydrolysates still needs technological improvements to achieve high purity levels with low-cost and less environmental impact. In this case, the comprehensive analysis encompassing the techno-economic–environmental aspects of the entire process from biomass reception to XOS production plays an important role in its development. However, it is worth noting that experimental results needed for TEEA calculations are often dependent on the xylan source, and the viability of XOS production should be considered in relation to its integration within a biorefinery. Therefore, to advance future research, significant improvements in specific steps and a holistic evaluation of the entire biorefinery are both essential, demanding that the process experimental optimization of yields in each operation work together with the modeling and TEEA to reach a feasible and scalable scenario.

## Figures and Tables

**Table 1:** Production of XOS from different biomass sources through different techniques.

Pretreatment	Approach	Conditions	Xylanase in the 2nd Stage	XOS Yield (%)	XOS (g/L)	Ref.
**Sugarcane Bagasse**						
Hydrothermal	SS	195 °C 10 min 10% solid load	--	45.2	9.2	[30]
Hydrothermal	TS	195 °C 10 min 10% solid load	Xylanase Novozymes NS22036	90.8	32.5	[30]
Hydrothermal	SS	160 °C 100 min 10% solid load	--	50.5	12.4	[31]
Acid	SS	Acetic acid 1% 100 °C 15 min 10% solid load	--	18.4	5.0	[18]
Alkaline	TS	KOH 10% 25 °C 20 h 10% solid load	Xylanase AfxynA from *Aspergillus fumigatus* FC-2-2	70.0	4.0	[32]
Alkaline	TS	NaOH 8% 40 °C 24 h 10% solid load	Endo-β-1,4-xylanase from *Pichia pastoris*	57.4	--	[33]
Organosolv	TS	Imidazole 160 °C 1 h 10% solid load	Xylanase from *Aspergillus niger*	30.0	6.1	[34]
Organosolv	TS	Ethanol 50% 170 °C 1 h 10% solid load	Xylanase Novozymes NS22036	89.5	33.3	[30]
Steam Explosion	SS	0.5% H_2_SO_4_ 1905 min	--	38.2	NR	[35]
**Sugarcane Straw**						
Alkaline	TS	KOH 24% + NaBH2 1% 35 °C, 3 h 8% solid load	Endoxylanase, α-L-arabinofuranosidase, and feruloyl esterase	40.4	NR	[36]
Steam Explosion	SS	Straw 80% moisture 200°, 10 min 10% solid load	--	35.2	8.0	[37]
**Corncob**						
Acid	SS	Tartaric acid 60 mM 170 °C 10 min 10% solid load	--	56.4	NR	[38]
Acid	SS	Acetic acid 60 mM 170 °C 10 min 10% solid load	--	6.2	NR	[38]
Acid	SS	HCl pH 2.7150 °C 30 min 20% solid load	--	22.5	13.71	[39]
Alkaline	TS	NaOH 1% 121 °C 40 min 10% solid load	*Paenibacillus xylanivorans* GH10 and GH11 xylanases and metatranscriptomic GH11 xylosidase	22.8	NR	[40]
Alkaline	TS	NaOH 16% 120 °C 45 min 10% solid load	Endoxylanase from *Trichoderma viride*	9.0	NR	[41]
Steam Explosion	TS	Corncobs~100 mm 196 °C 5 min	Thermostable xylanase from *P. thermophila* J18	28.6	8.5	[42]
**Brewers’ spent grain**						
Steam Explosion	SS	180 °C 10 min 25% solid load	--	75.1	NR	[43]
**Rice husk**						
Alkaline	SS	NaOH 6% 120 °C 45 min 10% solid load	--	44.4	17.4	[44]
**Coffee husk**						
Alkaline	SS	KOH 24% 35 °C 6 h 8% solid load	--	31.8	NR	[36]

SS: Single stage; TS: Two stages; NR: Not reported.

**Table 3:** Commercial prices of XOS available in the market.

Value	Information/Observation	Reference
Prices of XOS in the market		
USD 20.00/kg–USD 45.00/kg	Depending on its purity level and on its amount	Alibaba [96]
USD 22.50/kg	Depending on its purity level	Jain et al. [97]
USD 25.00/kg–USD 50.00/kg	Depending on its purity level	Santibáñez et al. [67]
USD 25.00/kg–USD 50.00/kg	Depending on its purity level	Zhao et al. [98]
USD 25.00/kg–USD 50.00/kg	Depending on its purity level	Brenelli et al. [37]
USD 28.00/kg	Depending on its purity level	Amorim et al. [4]
USD 235.00/kg	Powder, 70% XOS and 26% maltodextrin, sold in pills	Amazon [99]
USD 632.30/kg	Powder, 95% XOS, sold in pills of 500 mg/pill	Unic Pharma [100]
USD 3680.00/kg–USD 20,000.00/kg	Analytical uses—high purity of xylohexaose, xylopentaose, xylotetraose, xylotriose, and xyloniose for use in research	Megazyme [101]

**Table 4:** Economic performance of XOS production.

Economic Performance	Information	Pretreatment Conditions	Reference
Minimum XOS selling price			
3.43 USD/kg–7.50 USD/kg	80% XOS from *Miscanthus*	Extrusion and autohydrolysis 190 °C for 10 min with 1:3 solid/liquid ratio	Lan et al. [104]
4.03 USD/kg–8.97 USD/kg	90% XOS from *Miscanthus*		Lan et al. [104]
4.84 USD/kg–10.64 USD/kg	95% XOS from *Miscanthus*		Lan et al. [104]
2.51 USD/kg	Dedicated XOS production from brewers’ spent grains	Steam explosion (180 °C/10 min) followed by enzymatic hydrolysis	Swart et al. [105]
2.18 USD/kg	Xylitol and XOS co-production from brewers’ spent grains	Hydrothermal pretreatment (120 °C/15 min) followed by enzymatic hydrolysis	Swart et al. [105]
5.50 USD/kg	Annexed XOS production from sugarcane	SO_2_-catalyzed steam explosion (195 °C/5 min)	Heerden [108]
Production costs			
1.18 USD/kg	XOS from corn stover in the Portuguese case study	Hydrothermal pretreatment (210 °C, 1:8 solid/liquid ratio)	Lopes et al. [109]
1.56 USD/kg	XOS from wheat straw in Chilean case study		Lopes et al. [109]
USD 18.36/kg	XOS from brewers’ spent grains	Subcritical water hydrolysis in two sequential reactors	Sganzerla et al. [107]

**Table 5:** Global warming potential (GWP) emissions of XOS production from biomass.

CO_2_ Emissions per Kg of XOS	Scenario	Reference
9.21 kg CO_2eq/_kg	Annexed XOS production from sugarcane	Heerden [108]
11.39 kg CO_2eq/_kg	Annexed XOS and PDO co-production from sugarcane	Heerden [108]
4.21 kg CO_2eq_/kg	bioethanol and XOS co-production from the lignocellulosic residue barley straw from cereal cultivation and BSG	González-García et al. [111]
3.8 kg CO_2eq_/kg–5.5 kg CO_2eq_/kg	Co-production of cello-oligosaccharides and XOS using cellulosic substrates from sugarcane straw	Barbosa et al. [110]

## Data Availability

All generated data is presented in this manuscript.
